# Effectiveness of the indigent support policy on food insecurity in South Africa: Experiences from Matatiele Local Municipality

**DOI:** 10.1016/j.heliyon.2023.e19080

**Published:** 2023-08-12

**Authors:** Saul Ngarava

**Affiliations:** Copernicus Institute of Sustainable Development, Vening Meinesz Building A, 8a Princeton Avenue, 3584 CB Utrecht, Netherlands

**Keywords:** Household food in-access scale, Heckman two step model, Indigent support policy, Propensity score matching, South Africa

## Abstract

The indigent and societally vulnerable have compromised capacities to achieve their full welfare potential. This necessitates polices that can cushion them, such as the indigent support policy in South Africa. However, there is little acknowledgement on the welfare effects of community and contextually derived support policies. The study seeks an understanding of the effectiveness of the indigent support policy on food insecurity in Matatiele Local Municipality, South Africa, using a cross sectional survey of a purposively selected sample of 549 households. Food insecurity, determinants of awareness and beneficiation as well as effectiveness from the policy are assessed through the Household Food In-Access Scale (HFIAS), Heckman two step model and Propensity Score Matching (PSM), respectively. Households are found to be food secure, with awareness and beneficiation from the indigent policy being affected by duration of stay, employment status, location, tenure, total monthly income, monthly food expenditure and food insecurity status. To add, the indigent support policy has a positive impact on food security. In conclusion, there is food security partly due to indigent support with beneficiation affected by various socio-economic factors. There is need to compliment indigent support products to include food products and promote the policy to increase awareness. 10.13039/100014337Furthermore, there is need to capacitate and coordinate policy making to target food insecure households to augment and magnify the positive effects of indigent support.

## 1 Introduction

In the endeavor to end poverty, Sustainable Development Goal (SDG) 1.3 was developed and adopted, prescribing to the implementation of “nationally appropriate social protection system measures for all, including floors, and by 2030 achieving sustainable coverage of the poor and vulnerable” [1]. Regionally, continents such as Africa have ascribed to a 2063 Agenda that prioritizes inclusive social and economic development through a 20% reduction in food insecurity by 2023 [2] and progressively extend social protection coverage through the 2019 Abidjan Declarations [3]. However, Africa still lags behind with only 17% of its population receiving at least one social protection benefit compared to 47% globally [4]. Furthermore, 140 million Africans are still facing acute food insecurity, with a range of short to long term actions being implemented to cushion the poorest households, chief among them social protection [5]. Social justice can be enhanced by social protection which is crucial in guaranteeing an acceptable livelihood standard [6,7]. According to Devereux et al. [8] social protection are all the private and public initiatives for income and consumption transfers to the poor. Social protection activities generate economic outcomes related to improving welfare and accumulation [9]. This assumes that households are resource-poor, undermining their ability of asset accumulation and achieving welfare objectives of food security, health, and education outcomes. Social protection programs are there to stimulate asset accumulation, with varying multiplier effects such as enhanced productivity, higher incomes and welfare [9,10].

Even though South Africa has a social protection system, it is not comprehensive consisting of social assistance (old age grants, child care grants, disability grants, care dependency, social relief of distress and war veterans), social insurance (unemployment insurance, compensation funds for occupational injuries and road accident fund), and occupational and voluntary schemes (pension and provident funds, retirement annuities and medical schemes) [11,12]. None-the-less, it is effective in poverty reduction [6]. There were over 11 million beneficiaries of social grants by December 2021 in South Africa, mostly dominated by child support grant (70.5%) and old age grant (20.1%) [13]. In December 2022, the GoSA had paid out R1 billion (US$56 million) unemployment insurance with 87.6% of this pay out devoted to unemployment benefits, 8.8% for maternity benefits, 3.2% for dependents benefit and 2.4% for illness benefits [14]. One of the ways that has been utilized for social protection has been through the indigent support policy.

In the context of this study, indigency refers to households which earn a jurisdictionally low income so that they qualify for municipal service subsidy [15]. The jurisdictional characteristic of indigent support therefore determines the kind of support that households may receive, all determined by particular municipal characteristics and functioning in lieu of municipal capabilities in income generation. Some of the principles of indigent support in South Africa center along equity, sustainability, reasonable choice, inclusivity, implementability and social justice [15]. About 22% of the 17 million households in South Africa have been classified as indigent, with nearly half of these found in eThekwini. However, 44% of all household in the Eastern Cape Province are registered as indigent households, requiring some sort of assistance [16]. This provides a precarious situation for the 2.2 and 9.2 million South Africans in a state of food emergency and crisis, respectively [17], as well as 17.2% and 7.3% who have no access to water and electricity, respectively [18], and the 32.9% unemployment rate [19]. Globally, the term indigent has not been extensively adopted, however indicators such as homelessness (1.5%) [20], poverty (46.9%) [21] and unemployment (5.3%) [22] amongst others can paint a precarious situation for the poor who stand to benefit from poverty alleviation or cushioning support. Kuhlengisa et al. [23] acknowledge that indigent support is a powerful vehicle in achieving social justice.

In South African municipalities, indigents are the poorest in society, classified to receive free basic amenities. These group of people are characterized by a monthly income that is less than R1500 (US$83) [24]. There has been a steady increase in the number of indigent beneficiaries’ country-wide, with those in the Eastern Cape levelling out. The number of indigent policy beneficiaries has gradually increased in terms of free energy provision in the Eastern Cape, while for water it has been decreasing since 2011 (Appendix 1) [[25], [26], [27], [28], [29], [30], [31]].

The indigent policy is introduced to improve access to basic services with the endeavor to reduce household poverty [32]. This through bridging the gap between the rich and the poor, thereby achieving social justice [33]. The initial duration of the indigent policy is seven years (2005–2012), with objectives of providing basic refuse, energy, sanitation, water and housing services at local municipal level [34], and is part of the national policy also working in tandem with provincial policies which add free basic education, housing and health [32]. Local municipalities have the prerogative to outline their own indigent policies [35]. For instance, Cape Town provides free 50kwh electricity while Tshwane has highs up to 100kwh per month. eThekwini also has 300 L of daily free water, while Johannesburg bases free services on the poverty indices [35]. Equitable share is used to finance the indigent policy to assist poor households obtain services [36]. There are also food assistance programs instituted by the relevant municipalities. However, this dependent on the financial viability of the municipality [37]. To qualify for indigent support in Western Cape for instance, municipalities target household that have a joint monthly income and/or property value of less than R3,500 (US$192.12) and R300,000 (US$1,6467.60), respectively [36].

Various factors have been identified as affecting the awareness and utilization of indigent support and how it affects food security. Banks et al. [38] find that there is low awareness of the benefits of indigent support in Vietnam which affects access. This is also echoed by Khairullina et al. [39] in Russia and Grebe and Mubiru [40] in Uganda. In the Philippines, Etrata and Montemayor [41] conceptualise socioeconomic as well institutional factors affecting the awareness and utilization of indigent support. However, there is lack of empirical inquiry as to their effects. Factors such as age, gender, marital status, ethnicity and employment status are identified by Veerachamy [42] in India, while gender, household size and availability of complimentary services are identified by Mwangi [43] in Kenya. Niño-Zarazúa [44] identifies factors such as institutional capacity, financial viability and politics from a macro perspective in Sub Sahara Africa. Studies by Hidrobo et al. [45], Devereux and Nzabamwita [46] and 10.13039/100004421World Bank [47] identify that indigent support policies have improved food security through increased food consumption, dietary diversity and asset accumulation, with Devereux [48] advocating for it intersectionality with social justice. However, most of these studies lack empirical rigour. For instance, Hidrobo et al. [45] and World Bank [47] use a meta-analysis while Devereux and Nzabamwita [46] use literature reviews.

Authors such as Barry and Roux [49] identify awareness of the indigent support policy at household level in South Africa. However, Rodina and Harris [50] as well as Kuhlengisa [51] indicate limited awareness, with residents fearing that the policy might cease to exist. In addition, inaccessibility of the essential services such as water has consequences on food security. This through improving disposable incomes [51]. Most studies on the indigent policy focus on implementation challenges. 10.13039/100014337Furthermore, support is being affected by institutional challenges, lack of coordination and participation and poor infrastructural maintenance [33]. In South Africa, the indigent support policy is criticized for being generic and homogenous in each municipality having negative consequence on social justice, reinforcing socio-economic inequalities [51,52]; reactionary, especially provided that it is established on the eve of local government elections [53]; inefficient creating backlogs [51]; insufficient [54,55]; and costly, with a blurred line between social responsibility and affordability [37]. Despite the obvious welfare implications of indigent support on households, there is limited literature especially incorporating food security. This in lieu of the country's Constitution also indicating lack of food and clothing being indicative of indigency. However, implementation of food and clothing support is through grant provision, which is a national prerogative [32], with however very weak checks and balances on food expenditures and security. To address in tandem economic, environmental and social dimension leads to more effective indigent support delivery through social equity [53]. The emphasis of literature has been on energy [52,54,55] and water [51,53]. According to Kimemia and Annegam [34], Kimemia [35] and Mashapha [56] the indigent policy should focus not only on assistance and expansion on water and energy but should go beyond capacitating households to improve their welfare. There is need to upscale access of indigent support by identifying households that are also experiencing food insecurity through food parcels for instance Ref. [35]. Even though the study by HSRC [35] is particularly extensive across South Africa, there is no evaluation on the level of awareness of indigent support and the determinants thereof. Furthermore, there is no account as to how indigent programs can have direct and/or indirect effect on food security. In addition, most of the literature utilizes qualitative approaches also neglecting the impacts on food security [36,51]. The objective of the study is to assess the effectiveness of the indigent support policy on food insecurity, taking Matatiele Local Municipality in South Africa as a case. The study is significant in lieu of the fact that a fifth of the South African households have been deemed indigent, with nearly half in the Eastern Cape. Furthermore, the country is deemed micro-level food insecure. Even though indigent support is recognized in South Africa's municipal by-laws, it is still unclear as to the effect of this statute which also appears to be vague. The Municipal Systems Act merely state the establishment of indigent support policy without finer details of what its objectives are and how it will be implemented [57]. The current study will offer a reference point of what has been experienced with the implementation of the policy. Availing impact of indigent support can provide policy implications to improve coverage which can be augmented by targeted socio-economic circumstances. South African experiences can also be echoed throughout the region and in developing countries especially given that the country is the second largest economy on the African continent.

## 2 Methodology

### 2.1 Description of study site

The study is carried out in Matatiele Local Municipality (MLM) in the Eastern Cape Province, South Africa (Appendix 2). The local municipality is characterized by a predominately rural and struggling subsistence sector in the former Transkei and Ciskei which is surrounded by highly developed farm lands [58,59]. Matatiele sits on 4356.9 km^2^, accommodating a population of 219 448 with 48% being under the age of 18 and a 40% employment rate, as well as 22.1% of the household having a monthly income less than R2 000 [60,61]. Close to 18.7% of the people in MLM were in poverty in 2016 [59]. In Matatiele, 65.5% of the population get water from a regional or local service provider while 29% have no access to electricity. Food insecurity is identified a one of the threats to local economic development in the local municipality [59]. Matatiele Local Municipality has an indigent support policy with 15 760 registered households, supporting with “free basic electricity (up to 50 kWh per month), non-grid energy, and alternative energy as well as refuse removal” [59,61,62]. This also augmented by six kilos liters of water, provided by Alfred Nzo District Municipality. The MLM has also been involved in programs such as the Integrated Community Outreach Program (ICROP) which provides food parcels to poor households [63,64]. In addition, the municipality also entered into agreements with registered non-profit-organizations to establish food kitchens to enhance food security [65]. Matatiele Local Municipality is an ideal study site because it exhibits characteristics of a typical rural-low-income community with high levels of poverty and unemployment as well as historical injustices brought about by class struggles. It also provides indigent support in all areas of water, energy, and food.

### 2.2 Conceptual framework

**Fig. 1:**
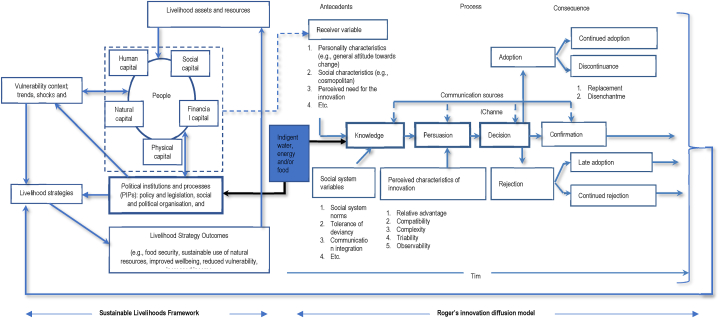
Conceptual framework.

**Source:** Adapted and modified from Chambers and Conway [69] and Rogers [70,71].

The study adapts a modified Rogers Innovation Diffusion Model (RIDM) embedded within the Sustainable Livelihoods Framework (SLF) (Fig. 1). The RIDM delineates the process followed by an individual in adopting an innovation or practice accounting for both the attributes of the user and the potential innovation or practice. The SLF on the other hand identifies challenges and management challenges to overcome them [[66], [67], [68]]. The diffusion innovation theoretical construct uses the following sequence in terms of utilizing or benefitting from a policy (i) knowledge (exposure to the policy), (ii) persuasion (creation of negative/positive perceptions), (iii) decision (deciding/not deciding to be a beneficiary of indigent support), (iv) implementation (actual utilization of the indigent support policy, and (v) confirmation (corroboration or rejection based on outcome in utilizing the policy) [66]. Contextualizing the current study, Fig. 1, shows that these decisions are informed by the social, political, and economic contexts in political institutions and processes (PIP) which set and roll out the indigent support policy to inform and have influence on livelihood strategies that can be pursued by individuals. The indigent support policy through the PIPs is informed by the livelihood assets (social, human, physical, financial, and natural) that surround underprivileged rural people, which are also bounded by vulnerability contexts, be it food insecurity, poverty, and climate change, amongst others. Interaction of the vulnerability contexts, livelihood assets and PIPs inform the livelihood strategies pursued by individuals, in turn conferring livelihood outcomes such as increased income, food security and sustainable resource utilization, amongst others. Thus, adoption of the innovation indigent support policy has direct and/or indirect effect on food security status of individuals based on the attributes of both the individual and policy, livelihood assets and vulnerability contexts.

### 2.3 Research design

Purposive sampling is used in the quantitative cross-sectional survey study. The Eastern Cape Province up to Matatiele Local Municipality are purposively selected. Ward, village, and household selection are also purposively sampled. The discriminatory selecting criteria and sampling frame comprise of households with water, energy and food insecurity, information of which is obtained from the Matatiele Spatial Development Framework Review [72]. The purposively selected households are also informed by households that are engaged by a local NGO in a broader project on “Water-Energy-Food nexus multi-actor governance for social justice”. The Yamane [73] method as shown in Eq. (1) is used in calculating the sample size.

(1) $$n=\frac{N}{1+N{\left(e\right)}^{2}}$$

where n is the sample size, N is the population, which is 18 642 households (from nine purposively selected wards), and e is the degree of accuracy, which is 95% in the study.

Proportional sampling is used to obtain the sample size from each ward (Appendix 3). The study uses a sample size of 549 households, with ward eight having a sample shortfall, whilst the others surpassing the targeted. The sample and the method used in sample selection are valid in a rural-poverty context, with the study site located in the former Transkei with high poverty and social injustice levels in the country. Thus, any findings are limited and applicable to poor rural households in the former homelands of South Africa.

### 2.4 Analytical framework

The initial analysis of the study uses the Household Food In-Access Scale (HFIAS) which is developed by the Food and Nutrition Technical Assistance (FANTA) project [74] to measure food insecurity. HFIAS utilizes a four-week recall questions, based on their behavioral and psychological experience, to measure food insecurity in nine questions. The summation of the responses to the HFIAS questions resulted in a score of 9≤HFIAS≤45 which is categorised into: 0–9 (food secure), 10–18 (moderately food secure), 19–27 (somehow food secure), 28–36 (food insecure), and (37–45) extremely food insecure.

The study goes on to utilize the Heckman two step model to assess determinants of awareness and utilization of the indigent support policy. The Heckman two step model is used because it accommodates both heterogenous and endogenous effects, reduces complicated modelling structures whilst retaining the benefits of single models [75]. In the current study, awareness and utilization of the indigent support policy is not discriminatory on whether it is from water, energy and/or food, but any of the three and any combination thereof. In both hurdles, Probit model is used, where the first hurdle is awareness of the indigent support policy while the second hurdle is the utilization of the indigent support policy. The Probit model is summarized in Eq. (2):

(2) $$Prob\left(I_{i}=1\;|X\right)=F\left(X_{i}\beta \right)$$

where I is the dependent binary variable of being aware or utilizing the indigent support policy, F(·) is the normal cumulative distribution function, $X_{i}$ is the set of explanatory variables. The general form of the Probit model is shown in Eq. (3) while the variables used are shown in Table 1.

(3) $$P_{I}=\beta_{0}+\beta_{1}\left(LOC\right)+\beta_{2}\left(AGE\right)+\beta_{3}\left(GEN\right)+\beta_{4}\left(ETH\right)+\beta_{5}\left(MARST\right)+\beta_{6}\left(EDUL\right)+\beta_{7}\left(DURST\right)+\beta_{8}\left(TEN\right)+\beta_{9}\left(EMPL\right)+\beta_{10}\left(HHS\right)+\beta_{11}\left(SOURCEINC\right)+\beta_{12}\left(TOTINC\right)+\beta_{13}\left(MONTHWAT\right)+\beta_{14}\left(MONTHEN\right)+\beta_{15}\left(MONTHFD\right)+\beta_{16}\left(HFIAS\right)+\beta_{17}\left(SOCFD\right)$$

where $P_{I}$ is the probability of being aware and/or utilizing the indigent support policy.

**Table 1:** Variables used in the Heckman two step model.

Variable	Explanation	Type of measurement	Expected sign
Dependent variables			
$I_{1}$	Hurdle 1	Nominal: 0-Awareness of the indigent support policy, 1-Otherwise	
$I_{2}$	Hurdle 2	Nominal: 0-Beneficiary of the indigent support policy, 1-Otherwise	
**Independent variable**			
LOC	Ward	Nominal: 0-Ward 3, 1-Ward 4, 2-Ward 5, 3-Ward 7, 4-Ward 8, 5-Ward 9, 6-Ward 11, 7-Ward 12, 8-Ward 26	−/+
AGE	Age of household head	Scale: Actual number in years	–
GEN	Gender of household head	Nominal: 0-Male, 1-Female	–
ETH	Ethnicity	Nominal: 0-Xhosa, 1-Sotho, 2-Zulu	−/+
MARST	Marital status of household head	Nominal: 0-Single, 1-Married (monogamous), 2-Married (polygamous), 3-Widow, 4-Widower, 5-Divorced, 6-Separated, 7-Living with partner	−/+
EDUL	Educational level of household head	Ordinal: 0-None, 1-Pre-School, 2-Primary, 3-Secondary, 4-Tertiary	–
DURST	Duration of stay in study area	Scale: Actual number in years	–
TEN	Tenure	Nominal: 0-Own, 1-Rent, 2-Family trust	+
EMPL	Employment status of household head	Nominal: 0-Unemployed, 1-Formal employment in non-agricultural related activities, 2-Formal employment in agricultural related activities, 3-Informal/self-employment in non-agricultural related activities, 4-Informal/self-employment in agricultural related activities	−/+
HHS	Household size	Scale: Actual number of persons	–
SOURCEINC	Main source of income	Nominal: 0-Formal employment in non-agricultural related activities, 1-Formal employment in agricultural related activities, 2-Informal/self-employment in non-agricultural related activities, 3-Informal/self-employment in agricultural related activities	+
TOTINC	Total monthly income	Scale: Actual amount in Rand	+
MONTHWAT	Monthly water expenditure	Scale: Actual amount in Rand	–
MONTHEN	Monthly energy expenditure	Scale: Actual amount in Rand	–
MONTHFD	Monthly food expenditure	Scale: Actual amount in Rand	–
HFIAS	Household food in-access scale	Scale: Truncated actual score	+
SOCFD	Source of food	Nominal: 0-Purchase, 1-Own production, 2-Gift, 3-Barter	−/+

The study further assesses the impact of the indigent support policy on food insecurity through Propensity Score Matching (PSM) as used by Belete and Bayu [76], Habib et al. [77] and Naveed et al. [78]. According to Shahidi et al. [79], PSM enables construction of well-matched control groups where there is no apparent comparisons. King and Nielsen [80] argue that PSM, and in particular nearest neighbor matching, is not ideal relative to other matching methods as efficiency is reduced and also increases imbalance and bias, while Guo et al. [81] argue to the contrary, that use of PSM is dependent upon the fit between data generation process and analytical model assumptions. Wang [82] actually argues that the problem does not lie in whether or not to use PSM, but rather when and how to use it. This obstacle is overcome by referring to other matching methods such as kernel and radius [83] since Guo et al. [81] highlight that King and Nielsen's [80] argument is not against the entirety of matching methods. Furthermore, Heinrich et al. [84] identify three data conditional requirements that need and where fulfilled by the study to use PSM, which include (a) access to a large number of variables correctly characterizing the propensity scores, (b) drawing data from the same source, and (c) a large enough pool of the treated group to compare. Choice of matching method depends on the goals of the analysis, and data quality. This has resulted in several matching methods which include nearest neighbor, optimal pair, optimal full, generalized full, genetic, exact, coarsened exact, subclassification and cardinality, amongst other [85]. The objective of the study is not to compare matching methods, but to apply based on the data quality and the goal of the analysis. According to Greifer [85], if the objective of the analysis ATE, then profile, subclassification, generalized full or optimal matching are ideal. If the objective is ATT, then any matching method can be used. The target inference of the study is ATE with a “good enough” data set and full matching is used. However, to check for robustness, diagnostics of nearest neighbor, kernel, radius, and stratified matching are also performed. Coarsened exact matching is not used because there were no continuous covariates. Since propensity scores are also used, Mahalanobis matching is also not ideal. Profile matching is also not ideal because the covariates did not have units ranging between zero and one. Generalized full matching or subclassification is not used as it requires very large data sets [85]. In the context of the current study, standard regressions are not ideal because of the presence of substantial differences between households that utilize and do not utilize indigent support. Difference-in-difference (DID) is also not ideal because the treatment of utilizing or not utilizing indigent support is not randomized [86], but rather based on socio-economic circumstances of household income. Other methods such as regression discontinuity design and regression double difference require pre and post intervention information [78], which is not covered by the current study. The PSM provides a framework to select comparable subgroups which utilize and do not utilize indigent support from the source population. In the PSM, for a household h, (where h=1…H and H denotes the population of households), the impact evaluation separates the impact of being a beneficiary of the indigent support policy ($I_{h}=1$) on a certain outcome [${HFIAS}_{h}\left(I_{h}\right)$] food insecurity from what would happen without being a beneficiary of the indigent support policy ($I_{h}=0$), the counterfactual. This the difference between the outcome of being a beneficiary of the indigent support policy for household h and the counterfactual potential before/without being a beneficiary of the indigent support policy (Eq. (4)).

(4) $$\tau_{h}={HFIAS}_{h}\left(1\right)-{HFIAS}_{h}\left(0\right)$$

The impact $\tau_{h}$ cannot be observed since a household is either a beneficiary of the indigent support policy or not, but never both. The next stage is to ascertain the average treatment effect of the treated (ATET) (Eq. (5)):

(5) $$\tau_{ATET}=E\left[\tau |I=1\right]=E\left[HFIAS\left(1\right)|I=1\right]-E\left[HFIAS\left(0\right)|I=1\right]$$

The resulting PSM estimator for ATET is generalized in Eq. (6):

(6) $$\tau_{ATET}^{PSM}=E_{\mathrm{Pr}\left(X\right)|I=1}\left\{E\left[HFIAS\left(1\right)|I=1,\mathrm{Pr}\left(X\right)\right]-E\left[HFIAS\left(0\right)|I=0,\mathrm{Pr}\left(X\right)\right]\right\}$$

In the PSM, a Probit model is used with variables in Table 2.

**Table 2:** Variables used in the Propensity Score Matching (PSM) model.

Variable	Explanation	Type of measurement	Expected sign
Outcome variable			
HFIAS	Household Food In-Access Scale	Ordinal: 0 -food secure, 1-moderately food secure, 2-somehow food secure, 3-food insecure, 4-extremely food insecure	
**Treatment variable**			
I	Beneficiary of the indigent support policy	Nominal: 0-Beneficiary of the indigent support policy, 1-Otherwise	
**Independent variable**			
GEN N	Gender of household head	Nominal: 0-Male, 1-Female	+
MARST	Marital status of household head	Nominal: 0-Single, 1-Married (monogamous), 2-Married (polygamous), 3-Widow, 4-Widower, 5-Divorced, 6-Separated, 7-Living with partner	−/+
EMPL	Employment status of household head	Nominal: 0-Unemployed, 1-Formal employment in non-agricultural related activities, 2-Formal employment in agricultural related activities, 3-Informal/self-employment in non-agricultural related activities, 4-Informal/self-employment in agricultural related activities	−/+
EDU	Educational level of household head	Ordinal: 0-None, 1-Pre-School, 2-Primary, 3-Secondary, 4-Tertiary	–
SOURCEINC	Main source of income	Nominal: 0-Formal employment in non-agricultural related activities, 1-Formal employment in agricultural related activities, 2-Informal/self-employment in non-agricultural related activities, 3-Informal/self-employment in agricultural related activities	+

Three assumptions underline the PSM [66,87].

(a) The balancing assumption ensures households having similar propensity scores have similar unobservable characteristics, irrespective of benefiting from indigent support (Eq. (7)).

(7) IꓕX|Pr⁡(X)

(b) Benefiting from indigent support is as good as random as depicted in the conditional independence assumption (Eq. (8)).

(8) Y(0),Y(1)ꓕI|X∀X

(c) Probability of benefiting from indigent support for each X vector is strictly within the unit interval for sufficient overlap for beneficiary and non-beneficiary characteristics, finding adequate matches (Eq. (9)).

(9) 0<[Pr(X)=Pr⁡(D=1|X]<1

## 3 Results

### 3.1 Descriptive statistics

Table 3 shows that 50.3% of the household heads are male. Close to 54.1% of the respondents are of the Xhosa ethnic group, relative to 38.1% and 7.8% who are Sotho and Zulu, respectively. Most of the respondents are married (42.8%), while only 0.7% are divorced. Nearly 51.9% of the respondents have primary education with only 3.1% are uneducated. More than a third (67.8%) of the household heads are unemployed, while 12.2% are informally employed in agricultural related activities. The main source of income is from social grants (67.8%), followed by formal employment in non-agricultural related activities (10.2%) and remittances (7.8%), respectively.

**Table 3:** Demographics and socio-economic characteristics of the respondents in the study area.

Variable		Frequency (n = 549)	%
Gender	Male	276	50.3
	Female	273	49.7
Ethnicity	Xhosa	297	54.1
	Sotho	209	38.1
	Zulu	43	7.8
Marital status	Single	139	25.3
	Married (monogamous)	235	42.8
	Married (polygamous)	13	2.4
	Widow	73	13.3
	Widower	29	5.3
	Divorced	4	0.7
	Separate	19	3.5
	Living with partner	37	6.7
Educational level	None	17	3.1
	Pre-school	23	4.2
	Primary	285	51.9
	Secondary	197	35.9
	Tertiary	27	4.9
Employment status	Unemployed	372	67.8
	Formal employment in non-agricultural related activities	44	8.0
	Formal employment in agricultural related activities	21	3.8
	Informal/self-employment in non-agricultural related activities	45	8.2
	Informal/self-employment in agricultural related activities	67	12.2
Main source of income	Formal employment in non-agricultural related activities	56	10.2
	Formal employment in agricultural related activities	34	6.2
	Informal/self-employment in agricultural related activities	23	4.2
	Remittances	43	7.8
	Social grant	372	67.8
	Other	21	3.8

### 3.2 Indigent support policy and household food insecurity

**Fig. 2:**
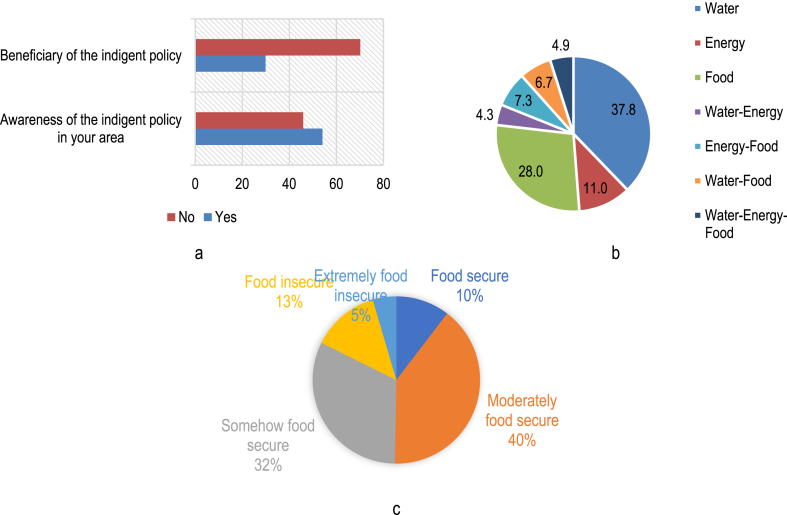
(a) Awareness and utilization of the indigent support policy, (b) sector from which household is a beneficiary of the indigent support policy, and (c) food insecurity status of households.

As indicated in Fig. 2(a), 54.1% of the respondents are aware of the indigent support policy, while 29.9% are beneficiaries of the indigent support policy. The households obtain support in the form of water (37.8%), food (28.0%) and energy (11%), respectively (Fig. 2(b)). However, 4.9% of the households are obtaining support in all three resources. Approximately 40.0% of the households are moderately food secure, while 32.0% are somehow food secure. Only 13% of the households are food secure, while 5% are extremely food insecure (Fig. 2(c)).

### 3.3 Awareness and determinants of using the indigent support policy

Duration of stay in the area (5% level) and employment status (1% level) are significant variables in the awareness of the indigent support policy while location (10% level), tenure (5% level), total monthly income (1% level), monthly food expenditure (1% level) and the food insecurity status (1% level) are significant factors in being a beneficiary of the indigent support policy (Table 4). The overall model is significant at the 10% level. The inverse mill ratio is negative and insignificant. This is due to the fact that some of the variables in the first hurdle might not be available in the second hurdle [88].

**Table 4:** Determinants of awareness and utilization of the indigent support policy.

	Awareness of indigent support policy				Beneficiary of indigent support policy			
Variable	β	Std. Err.	z	P>|z|	β	Std. Err.	z	P>|z|
Location (Ward)	−0.003	0.009	−0.35	0.728	−0.019	0.010	−1.94	0.052
Age	−0.003	0.003	−1.06	0.291	0.004	0.006	0.67	0.502
Gender	0.036	0.801	0.44	0.657	−0.122	0.135	−0.90	0.368
Ethnicity	0.051	0.079	0.65	0.517	−0.160	0.104	−1.54	0.124
Marital status	0.032	0.023	1.38	0.168	−0.038	0.033	−1.15	0.252
Educational level	−0.091	0.062	−1.46	0.143	0.104	0.095	1.09	0.275
Duration of stay	−0.004	0.002	−2.01	0.044	0.003	0.003	0.77	0.440
Tenure	0.116	0.103	1.12	0.264	−0.224	0.102	−2.20	0.028
Employment status	−0.081	0.025	−3.16	0.002	0.029	0.046	0.62	0.534
Household size	0.0004	0.014	0.03	0.975	−0.017	0.025	−0.67	0.500
Main source of income	−0.005	0.028	−0.17	0.868	0.032	0.051	0.62	0.538
Total monthly income	0.00003	0.00004	0.96	0.339	−0.00007	0.00002	−2.91	0.004
Monthly water expenditure	−0.0001	0.0005	−0.25	0.804	0.0007	0.001	0.68	0.494
Monthly energy expenditure	−0.00009	0.0002	−0.46	0.644	0.0003	0.0004	0.91	0.361
Monthly food expenditure	−0.00007	0.0001	−0.54	0.592	0.0003	0.0001	2.66	0.008
HFIAS	−0.097	0.091	−1.06	0.289	0.236	0.080	2.97	0.003
Source of food	0.131	0.115	1.14	0.256	0.168	0.227	0.74	0.459
Constant	1.608	0.679	2.37	0.018	−0.142	0.522	−0.27	0.786
Summary statistics								
$\chi^{2}$	26.05							
$P_{value}$	0.074							
Mills lambda	−0.736	0.807	−0.91	0.362				
rho	−1.096							
sigma	0.672							

An increase in the duration of stay in the area results in the household being aware of the indigent support policy. The results further indicate that as the employment status becomes more informal/self-employment, the more likely the households become aware of the indigent policy. There is differentiated beneficiation from indigent support based on the different wards. Households that are renting and are on family trust are more likely to be beneficiaries of the indigent support policy. Family trust in the context of the current study refers to individuals or a household that is living on the premise entrusted by their extended family members. Household with higher food expenditures and are more food insecure are more likely to be beneficiaries of the indigent support policy.

### 3.4 Impact of the indigent support policy

The results show that a household that is a non-beneficiary of the indigent support policy has a higher HFIAS score of 0.226 compared to a household that is a beneficiary of the indigent support policy (Appendix 4). This indicates that the indigent policy has a positive impact on food security, which is significant at the 5% level. It is also worth noting that the HFIAS measure is categorical and hierarchical, indicating equal distances between two categories, and thus a 0.226 change would translate into a 22.6% change. Thus, being a beneficiary of the indigent policy increases the food security status by 22.6%.

The matching methods in Appendix 5 confirm the earlier results of the indigent policy positively influencing the food security status of the households. There is significant impact which varies between 13.4% and 22.6%.

**Fig. 3:**
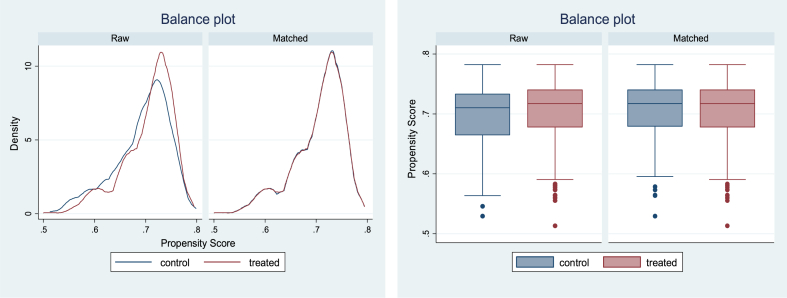
Kernel density and box plot showing the raw and unmatched control and treated groups.

The covariate summary of results shows that the PSM model reduces the standardized differences in the raw data compared to the matched (Appendix 6). The kernel density and box plot show that balance is achieved after matching, and thus the impact assessment is robust (Fig. 3).

## 4 Discussion

The result show that there is relatively average level of awareness and lower levels of beneficiation form indigent support. A study by Barry and Roux [49] also finds that even though there is awareness of the indigent policy, there is low usage. However, Barry and Whittal [89] also find that households are aware of the indigent policy and are making use of it. Even though Rodina and 10.13039/100016279Harris [50] find limited awareness of the indigent support program, even amongst marginalized urban communities, Schultz and Hendrickse [36] indicate that awareness of the indigent program is improved through processes such as a budgetary process encouraging public participation, public library notices, pamphlets, print and electronic media and by staff presentations in ward committees. Low levels of awareness will result in the low utilization of indigent support [[38], [39], [40]], and result in lower welfare effects. However, Pillay and Mutereko [24] indicate that awareness should not only be the purview of the beneficiaries, but also the implementers of the policy. Their study finds that indeed, the implementers are aware of the policy and understood its objectives. However, execution is also another factor in the implementation phase which is lacking. In addition, implementation is marred by inefficiencies and promotion of dependencies. For instance, beneficiaries interchange between qualifying and being part of the program, and exiting, only to be registered again, causing a logistic nightmare. This is also in conjunction with lack of documentation to prove that a household qualifies to be called indigent [24]. The policy therefore requires a clear entry and exit strategy and a broad spectrum of qualification for a household to be called indigent. This will go a long way in improving the utilization of the support to “deserving” households. In addition, the indigent policy is also discriminatory as it targets certain levels of indigents, usually with access to amenities such as municipal water and electricity. The very poor and ideally the most indigent who have no access to these amenities are marginalized and left in the fringes. This can have a bearing on the awareness and subsequent utilization of the support policy.

The results further show that awareness and beneficiation from indigent support is dependent upon variables such as duration of stay, employment status, location, tenure, monthly income, and food expenditures. Barglowski and Bonfert [90] highlight that duration of stay shapes social security practices. Ye et al. [52] aver that there is an association between household formation and dissolution with access to electricity [91]. Household formation and dissolution results from duration of stay and has implications on lived experiences [92]. Generational knowledge improves when households live in an area for longer periods of time. This knowledge can include actionable information in terms of indigent support. This tends to lower the perception risks [93]. However, the intrinsic knowledge gained by staying in area for long periods and the formal and informal relationships built, can reduce efficiencies in implementing the indigent support program. This through creation of opportunities in patronage, political interference, corruption, nepotism, and fraud, amongst others. This will ultimately affect the awareness and utilization of indigent support by the community.

Deficits in social protection closely associate with informal employment [94]. Informal/self-employment associates with low and inconsistent incomes which compromise the food security and welfare of households. This increases the likelihood of households having the knowledge and awareness of indigent support. This quite significant in South Africa especially given that informal employment is a third of the country's employment [11]. However, this reinforces the poverty cycle as the informality and/or increase in unemployment strains social and economic interventions with households becoming increasingly dependent upon such interventions such as indigent support [6]. 10.13039/100014337Furthermore, to keep benefitting from the indigent support, you need to remain informally employed or unemployed. This tends to breed rent seeking behavior in the short to medium term and free riders in the long term (as this based on public resources), which perpetuates the poverty cycle and the financial and economic strain affecting sustainability of the indigent support policy.

Food, energy, and water insecurity are not homogenous. Thus, there will be differentiated needs from the various localities. Lack of resources can limit adaptation to food and water insecurity. Jorgensen and Siegel [95] aver that different localities exhibit varied levels of poverty, hence have varied choices, needs, and wants from social protection. This based on heterogenous assets, income levels, risks, and economic/social exclusions, amongst others. Locational differences also have effect on individual behaviors, aspirations and expectations [95]. Devereux and White [96] go on to indicate that authorities responsible for the design, finance and delivery of social protection play a leading role in the biases based on location, duration, and scale. This through differentiated agency and power which is also locationally and heterogeneously based. It is the influential and their locales that tend to benefit most from developmental policies through patronage, political interference, corruption, nepotism, and fraud, reinforcing social injustice. This also translated into the welfare outcomes of water, energy, and food security. Thus, due to heterogeneity in locations, there will be varied levels of awareness and utilization of indigent support. Differentiated needs and wants can be tackled through inclusive representation in indigent support policy formulation. Platforms such as ward committees and Integrated Development Plan (IDP) forums which allow community representation in local government decision making offer platforms to include different locational representations, thereby allowing social justice (through distributive justice) access to energy security support which translates into food security.

Tenure is associated with awareness of indigent support policy as alluded to by Barry and Roux [49]. In this instance, homeowners effectively keep their municipal payments up to date to be issued with clearance certificates to effectively benefit from indigent support. Household that are renting and on family trust do not own much of the assets. Their adaptability is compromised hence reliant upon indigent support. Furthermore, ownership to land is essential for poverty alleviation [97]. It is a base for food production and water availability, influencing food and water security. Brown et al. [98] went on further to indicate that it provides an income guarantee. Lack of access to land can compromise these, forcing people to seek for alternatives through indigent support for instance. Equitable access to land therefore becomes imperative to reduce food and water insecurity as well as the burden on indigent support. However, Mahadevia [97] argues that providing tenure security without other securities will not enable the poor to retain them.

More food insecure households become more aware and benefit from indigent support when there is high food expenditure because of reduction in disposable income which can be dedicated to food. Pradhan [99] indicated that the high food expenditures of households make them susceptible to price shocks, making it key that they become aware and benefit from alternative support. Food insecure households are dependent on indigent support to augment their food supplies. However, Jahangir et al. [100] indicates that support can actually promote insecurity through promotion of non-nutrient rich food. This through the enhanced disposable incomes enabling purchase of other food items.

The positive impact of indigent support on food security is consequent of free electricity which is found by Ye et al. [52] to increase access to electricity. This will in turn improve food security as energy for cooking purposes is made available, as well as energy expenditure is diverted to food purchases. The increase in disposable incomes improve quantity and quality of food, as well as asset accumulations including livestock [45]. Osabohein et al. [101] also find that social protection improves food security in West Africa. The study is however focusing on the macroscale. Similar findings are made by Gilligan et al. [102] in Ethiopia. Hidrobo et al. [45] find that social protection improves food expenditure by 13% and caloric acquisition by 8% mainly availed through access of disposable incomes in developing countries. However, Devereux and Nzabamwita [46] highlights of inconsequential individual nutritional outcomes through under-coverage and low values, and Devereux [48] advocates for support that enhances productive base through agriculture or stabilizes income rather than handouts that create a free-rider syndrome [103,104]. The findings have a bearing on the indigent policy as it shows multiplier effects beyond the intended energy subsidy to other welfare outcomes of food security. It also shows the household-level nexus and complementarity as well as trade-offs that exists between energy and food supply. In indigent households, this complementarity exists when energy is used for processing (meal preparation) instead of food production. Trade-offs are exhibited when there is budget or expenditure transfer from energy to food which is induced by indigent support of free energy. This confirms the synergies and trade-off that exist in energy and food as alluded to by Vahabzadeh et al. [105].

## 5 Limitations

The study limitations can be viewed from a conceptual, spatial, temporal, and methodological (internal and external validity) standpoint. Conceptually, indigent support in the current study is “loosely” defined as subsidizing household's basic amenity needs. However, based on the definition of indigent household, other support structures such as social protection can also be deemed indigent support. Spatially, the study is limited to Matatiele Local Municipality. Temporally, the study is a cross sectional survey. Internal validity is achieved through the design and analysis which have been used before in literature and are pertinent to answer the question on impact and effectiveness. 10.13039/100014337Furthermore, the PSM analytical framework relied on assumption of conditional independence, however there are confounders influencing both beneficiaries and non-beneficiaries of indigent support. External validity and generalization to other contexts is limited due to spatial and temporal heterogeneity.

## 6 Conclusion and recommendation

The study concludes that there is a certain degree of awareness, and lesser still, of benefiting from the indigent support program determined by various socio-economic variables resulting in improved food security. The study recommends improving the bundle of social protection products to include food security. This can be through active promotion of food parcels at household level in conjunction with the school feeding program which can aid in reducing food insecurity. Municipalities should actively promote beneficiation of indigent support to go beyond mere awareness through targeting electronic and print media which is accessible to households. Municipalities need to be proactive and capacitate themselves through real time interactive technologies such as mobile applications that track indigent households and make it easier to register new ones. To increase effectiveness, the indigent support should target households that have low incomes, involved in informal employment in lieu of their food expenditures. Thus, support should not be a one size fit all, but should be tailor made for household needs. There is need for interdepartmental coordination especially between local municipalities and Department of Social Development on food security, which will improve efficiency in identifying, registering, and implementing indigent support. The study recommends conducting further studies targeting urban areas who have differentiated socio-economic circumstances for comparability purposes to provide a more nuanced picture of the effect of indigent support on food security. Further studies that use other matching methods can also be carried out to authenticate the study findings.

## Declaration of competing interest

The authors declare that they have no known competing financial interests or personal relationships that could have appeared to influence the work reported in this paper.

## Footnotes

Appendix A Supplementary data to this article can be found online at https://doi.org/10.1016/j.heliyon.2023.e19080.
