# Supplementary material for: Effectiveness of the indigent support policy on food insecurity in South Africa: Experiences from Matatiele Local Municipality
Source: Heliyon. 2023 Aug 12;9(8):e19080. doi: 10.1016/j.heliyon.2023.e19080 (PMC10457532; doi:10.1016/j.heliyon.2023.e19080)
Supplement: Multimedia component 1 [file mmc1.docx]

|  |  |
| --- | --- |
| 1. Number of indigent household beneficiaries in South Africa and the Eastern Cape Province | 1. Number of indigent household beneficiaries in the Eastern Cape Province and service beneficiation |

**Appendix 1:** Beneficiaries of indigent support in South Africa and the Eastern Cape Province

**Source:** StatsSA [16–22]
