# Supplementary figures and images for: Effectiveness of the indigent support policy on food insecurity in South Africa: Experiences from Matatiele Local Municipality
Source: Heliyon. 2023 Aug 12;9(8):e19080. doi: 10.1016/j.heliyon.2023.e19080 (PMC10457532; doi:10.1016/j.heliyon.2023.e19080)

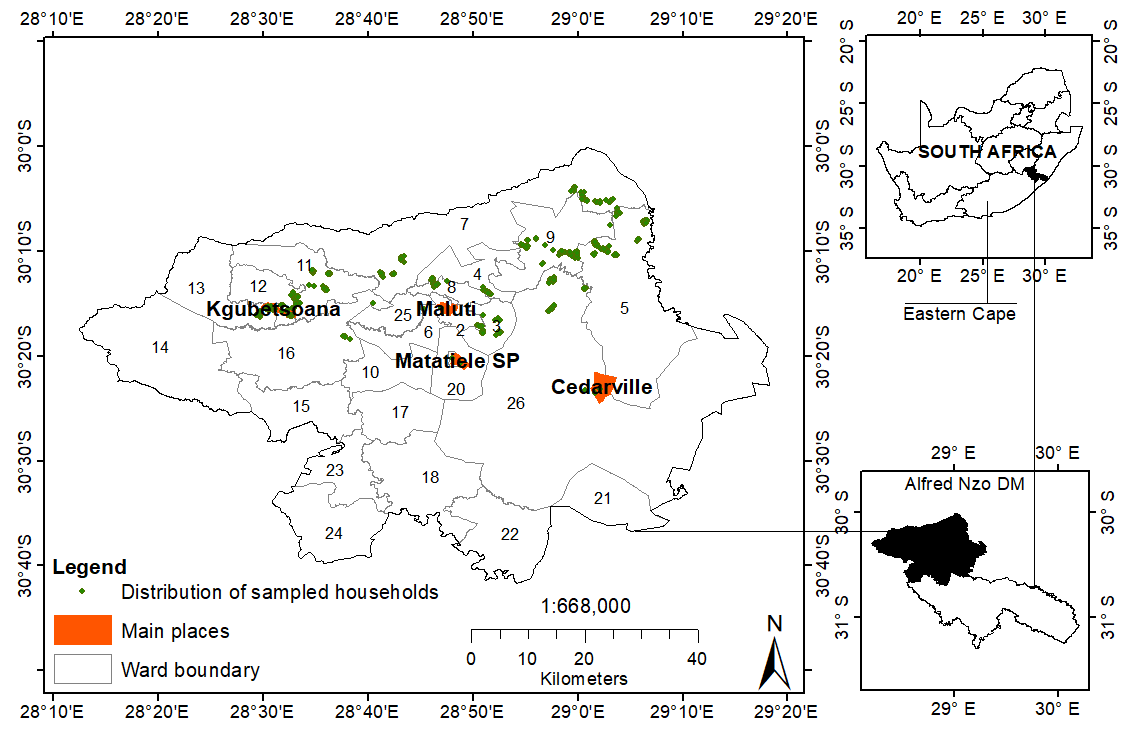


**Appendix 2:** Study area

Supplement: Multimedia component 2 [file mmc2.docx]
