# Supplementary material for: Effectiveness of the indigent support policy on food insecurity in South Africa: Experiences from Matatiele Local Municipality
Source: Heliyon. 2023 Aug 12;9(8):e19080. doi: 10.1016/j.heliyon.2023.e19080 (PMC10457532; doi:10.1016/j.heliyon.2023.e19080)
Supplement: Multimedia component 3 [file mmc3.docx]

**Appendix 3:** Sample size

| **Ward** | **3** | **4** | **5** | **7** | **8** | **9** | **11** | **12** | **26** | **Total** |
| --- | --- | --- | --- | --- | --- | --- | --- | --- | --- | --- |
| Population of households | 1752 | 2032 | 2022 | 2047 | 2279 | 2187 | 2091 | 1690 | 2542 | 18642 |
| Proportion | 0.09 | 0.11 | 0.11 | 0.11 | 0.12 | 0.12 | 0.11 | 0.10 | 0.14 | 1 |
| Sample size sought | 37 | 43 | 42 | 43 | 48 | 46 | 44 | 36 | 53 | 392 |
| Sample size actualised | 38 | 50 | 71 | 77 | 43 | 80 | 80 | 55 | 55 | 549 |
| Difference | 1 | 7 | 29 | 34 | -5 | 34 | 36 | 19 | 2 | 157 |
