# Supplementary material for: Effectiveness of the indigent support policy on food insecurity in South Africa: Experiences from Matatiele Local Municipality
Source: Heliyon. 2023 Aug 12;9(8):e19080. doi: 10.1016/j.heliyon.2023.e19080 (PMC10457532; doi:10.1016/j.heliyon.2023.e19080)
Supplement: Multimedia component 4 [file mmc4.docx]

**Appendix 4:** Impact of utilising indigent resources on food insecurity

| HFIAS Category | $\beta$ | Std Err. | $z$ | $P>\left\vert z \right\vert$ |
| --- | --- | --- | --- | --- |
| Are you a beneficiary of the indigent support policy? (No vs Yes) | 0.226 | 0.111 | 2.03 | 0.043 |
