# Supplementary material for: Effectiveness of the indigent support policy on food insecurity in South Africa: Experiences from Matatiele Local Municipality
Source: Heliyon. 2023 Aug 12;9(8):e19080. doi: 10.1016/j.heliyon.2023.e19080 (PMC10457532; doi:10.1016/j.heliyon.2023.e19080)
Supplement: Multimedia component 5 [file mmc5.docx]

**Appendix 5:** Matching results

| Matching | Number of treated | Number of control | ATET | Std. Err | t | % bias |
| --- | --- | --- | --- | --- | --- | --- |
| Nearest neighbour | 385 | 138 | 0.226 | 0.047 | 4.763 | -0.071 |
| Radius | 385 | 164 | 0.155 | 0.076 | 2.043 | 0.063 |
| Kernel | 385 | 164 | 0.147 | 0.066 | 2.234 | -0.002 |
| Stratified | 385 | 164 | 0.134 | 0.040 | 3.352 | -0.031 |
