# Supplementary material for: Effectiveness of the indigent support policy on food insecurity in South Africa: Experiences from Matatiele Local Municipality
Source: Heliyon. 2023 Aug 12;9(8):e19080. doi: 10.1016/j.heliyon.2023.e19080 (PMC10457532; doi:10.1016/j.heliyon.2023.e19080)
Supplement: Multimedia component 6 [file mmc6.docx]

**Appendix 6:** Covariate summary results

|  |  |  | Raw | Matched |
| --- | --- | --- | --- | --- |
|  |  | Number of observations | 549 | 770 |
|  |  | Treated observations | 385 | 385 |
|  |  | Control observations | 164 | 385 |
|  | Standardized differences | | Variance ration | |
|  | Raw | Matched | Raw | Matched |
| Gender | -0.060 | 0.073 | 0.997 | 1.010 |
| Marital status | -0.208 | -0.029 | 0.739 | 0.893 |
| Educational level | 0.033 | -0.065 | 0.958 | 1.044 |
| Employment status | -0.012 | 0.064 | 1.060 | 1.152 |
| Main source of income | 0.045 | 0.050 | 0.985 | 0.948 |
